# Pharmacotechnical aspects of a stable probiotic formulation toward multidrug-resistance antibacterial activity: design and quality control

**DOI:** 10.1186/s12906-023-04224-0

**Published:** 2023-10-31

**Authors:** Farkhonde Karimi, Amir Azadi, Navid Omidifar, Nima Montazeri Najafabady, Fatemeh Mohammadi, Radmehr Kazemi, Ahmad Gholami

**Affiliations:** 1 Biotechnology Research Center, Shiraz University of Medical Sciences, Shiraz, Iran; 2 Department of Pharmaceutical Biotechnology, School of Pharmacy, Shiraz University of Medical Sciences, Shiraz, Iran; 3 Pharmaceutical Sciences Research Center, Shiraz University of Medical Sciences, Shiraz, Iran; 4 Department of Pharmaceutics, School of Pharmacy, Shiraz University of Medical Sciences, Shiraz, Iran; 5 Department of Pathology, School of Medicine, Shiraz University of Medical Sciences, Shiraz, Iran; 6 Endocrine and Metabolism Research Center, Shiraz University of Medical Sciences, Shiraz, Iran

**Keywords:** Antibacterial activity, Antibiotic resistance, Probiotics, Topical gel formulation, MRSA, VRE

## Abstract

As a well-known group of the probiotic family, the Lactobacillus has increasingly contributed to hindering the growth of pathogens, particularly resistant species, in the last decades. Since antibiotic resistance has become a severe problem in global healthcare systems and considerably increased the mortality and morbidity rate in infectious diseases, we aimed to obtain a new stable formulation of Lactobacillus to overcome resistant infections. For this purpose, we designed various gel formulations containing *Lactobacillus rhamnosus* (*L. rhamnosus*) as an active pharmaceutical ingredient (API) in a water base and oil base gel, evaluated the probiotic stability in formulation to obtain an optimum formulation, and finally, investigated the antibacterial activities of that against two common hospital-associated multidrug-resistant pathogens, methicillin-resistant Staphylococcus aureus (MRSA) and vancomycin-resistant Enterococcus (VRE). Furthermore, the pharmaceutical aspects of the optimum formulation, including stability, homogeneity, spreadability, pH value, conductivity, and rheological behavior, were assessed.

The results indicated that the optimum formulation based on glycerol exhibited desirable pharmaceutical properties, including long-term stability, a perfect level of homogeneity, an acceptable range of spreadability with pseudo-plastic thixotropic behavior, and a promising antibacterial potential against MRSA and VRE. Our findings indicate that this novel probiotic formulation could be an excellent candidate to cope with antibiotic-resistant species, representing a hopeful treatment potential for topical applications, particularly in incurable infections. However, further in vivo studies seem warranted to evaluate their bactericidal activity against multi-drug resistant microorganisms.

**Supplementary Information:**

The online version contains supplementary material available at 10.1186/s12906-023-04224-0.

## Introduction

Antibiotic resistance, a significant challenge in the healthcare systems and one of the leading causes of medical treatment's increasing costs and complexity has attracted ever-increasing attention in recent studies [1]. Methicillin-resistant Staphylococcus aureus (MRSA) and vancomycin-resistant Enterococcus (VRE) are the known hospital-acquired microorganisms that are typically isolated from human samples such as infected urine, Cerebrospinal fluid (CSF), feces, and tissues [2, 3]. These pathogens could potentially increase morbidity and mortality rates due to their intrinsic and acquired resistance to multiple antibiotics [4, 5]. Moreover, these resistant bacterial infections remarkably prolong the inflammatory phase and the elevation of pro-inflammatory cytokines in the wound healing process, leading to the wound's chronic state and deferring complete treatments [6–8].

The health benefits and prophylactic potentials of probiotics, mainly lactic acid bacteria, have been noticed in the last two decades as they can improve immune system responses and cope with antibiotic microbial resistance in different infectious diseases, such as gastrointestinal, respiratory, and urinary infections [9–11]. Furthermore, an overview of previous studies showed that these strains particularly *Lactobacillus rhamnosus (L. rhamnosus)* pose antiadhesive and anti-biofilm activities and inhibitory effects against yeast and bacterial colonization [12, 13]. *L. rhamnosus* also revealed a significant reducing potential of cytotoxicity associated with MRSA and VRE infections. It prevented the binding of these resistant pathogens to the host cells as an immunogenic mechanism [14–16], all of which make Lactobacillus bacteria suitable candidate for probiotics therapy to overcome the infections [17].

The conventional formulations of probiotics were based on multispecies probiotics, most of which were administered for treating a wide range of gastric-related diseases. However, the next generation of probiotics formulations focuses on single-strain probiotics to achieve their effect [18–20].

In other hand, as systemic antibiotics' frequent and long-term use has remarkably increased microbial drug resistance, topical antimicrobial agents could be a good choice of systemic antibiotic alternatives, as suggested in previous findings [21]. Although topical probiotic therapy has recently appealed to many study interests, the pharmacotechnical aspects have been less considered, and the most have studied on the lysate, supernatant, or spore of probiotic bacteria have been used. Barthe M et al. evaluated the wound healing effects of a topical mixture of some probiotics spores in a chemical matrix and just paid on healing effects. In another study, the wound healing potential of a topical gel containing the fresh form of Lactobacillus plantarum was investigated [22–24].

According to our knowledge, no reports have evaluated a topical formulation containing viable *lactobacillus rhamnosus* for therapeutic applications. therefore, we aimed to develop a novel probiotic formulation for topical administrations which contained *lactobacillus rhamnosus* species with favorable stability by the time. Furthermore, pharmaceutical properties of formulation were precisely characterized to be able to considered as a medical product. For this purpose, we designed various probiotic gel formulations containing live *L. rhamnosus* based on oil and water to achieve desirable bacterial stability and gel formulation quality formulation. The bacterial stability test was carried out for all groups. The most stable formulation was further evaluated regarding pharmaceutical properties, including formulation stability, homogeneity, rheology, spreadability, pH, and conductivity assay. Furthermore, the optimum-designed probiotic formulation's antibacterial activities were studied against MRSA and VRE, two resistant hospital-associated microorganisms isolated from human specimens.

## Materials and methods

### Materials

*Lactobacillus rhamnosus* (*L. rhamnosus*) IBRC_M10754 was purchased from the Iranian Biological Resource Centre. All raw materials listed as follows were purchased from Sigma Chemical Co. (St. Louis, MO): De Man, Rogosa and Sharpe agar (MRS) medium, agar, glycerol (C_3_H_8_O_3_), olive oil, carboxymethyl cellulose (CMC), carbomer, HEPES (4-(2-hydroxyethyl)-1-piperazineethanesulfonic acid) and Mueller–Hinton broth. Ringer's solution and sodium chloride (NaCl) were purchased from Samen Pharmaceutical Co., Iran.

### Formulation development

In this study, we designed five different formulations in two main groups: water base and oil base gels. In water-based formulations, sodium chloride, Ringer's solution, and HEPES buffer were used as a solvent, and in oil-based formulations, glycerol and olive oil were applied. Also, carbomer and sodium carboxymethyl cellulose were used in aqueous formulations, and polyethylene glycerol 400 and 4000 were used as gelling agents in oily formulations [25] (Table 1).

**Table 1:** The composition of designed formulations, water- and oil-based gel

Formulation	Gel Base	Solvent	Sodium carboxymethyl cellulose (% w/v)	Carbomer (% w/v)	PEG400 (% w/v)	PEG4000 (% w/v)
F1	W	Sodium chloride 0.9% w/v	2	1	-	-
F2	W	Ringer's solution	2	1	-	-
F3	W	HEPES buffer1M (4-(2-hydroxyethyl)-1-piperazineethanesulfonic acid)	2	1	-	-
F4	O	Glycerol 46.15% w/v	-	-	46.15	7.7
F5	O	Olive oil 46.15% w/v	-	-	46.15	7.7

*F* Formulation, *W* Water base gel, *O* Oil base gel

After the preparation of formulations, a particular concentration of *L. rhamnosus* precipitate was added to each group with a colony forming unit (CFU) of 1 × 10^9^ CFU/ml as an active pharmaceutical ingredient (API), pharmaceutical assessments were performed to study the quality of the designed gel formulations.

### Pharmaceutical assays

#### Formulation stability and viability of probiotic contents

The stability of *L. rhamnosus,* the probiotic species included in the designed gel formulation, was carried out in five formulations and solvents individually containing bacteria. Formulated gel bases without bacteria were used as the control group for each formulation. For this purpose, *L. rhamnosus* was grown in MRS broth to reach a stationary phase and maximum concentration of bioactive metabolites at 37 °C [26]. After 24h incubation, bacteria were collected by centrifugation (4000 rpm, 15 min) and added into each formulation (OD_600nm_ = 0.8, CFU: 1 × 10^9^). Then, all groups were inoculated on MRS Agar plates containing 1.5% Agar at 37 °C using serial dilution. After 48 h incubation, the count of the colonies on plates was reported, and CFU was calculated on days 1, 7, and 14 in all groups, which were stored at 8 °C. After the bacterial stability assay, the optimum formulation, which revealed the most stability on the 14^th^ day, was chosen for the pharmaceutical assay.

In addition, bacterial stability and organoleptic properties of the optimum formulation were evaluated at temperatures of 8 and 25 °C for 30 days as described by Albaayit SFA et al. [27]. For this purpose, physical changes, including color and odor, volume, breaking of suspension, crystal growth, and shrinking of the formulation, were evaluated visually on days 1, 7, 14, and 30 after preparation at both temperatures.

### Homogeneity assay

The homogeneity of the optimum formulation was evaluated, as reported in previous studies [28]. Accordingly, 5 g of the optimum formulation was centrifuged for 15 min at 3500 rpm to observe the possibility of phase separation. In this regard, homogeneity of the formulation was qualitatively classified as very good (no phase separation), good (appearance of supernatant in small volume), regular (phase separation with the appearance of slightly clotted), and poor (phase separation with the appearance of the pellet).

### Rheological assay

To evaluate the rheological behavior of the optimum formulation under different stresses, the viscosity was studied at 25 °C, 24 h after gel preparation, using a Cone and Plate viscometer (Brookfield R/S Plus Rheometer, Harlow Essex, United Kingdom).

### Spreadability assay

The spreadability of the optimum formulation was studied by mimicking the extensometer using two glass plates. The lower plate held the sample, on which 0.5 g of the formulation was placed, and the upper plate was responsible for exerting force on the sample through its weight (42 g) for 3 min [29]. Furthermore, to increase the exerting force on the sample, a known weight of 200 and 500 g was added to the upper plate, and then, the circle area and circle diameter of spreading samples were measured in three weights. Each test was carried out three times at a temperature of 25 °C.

### pH measurements and conductivity

The pH value and conductivity of the optimum formulation, which was stored at two temperatures of 8 °C and 25 °C, were measured individually on days 1, 7, 14, and 30 using a pH meter (Sartorius PB-10, Germany). The formulations were diluted in 1:9 ratios by deionized water before assessment [30].

### Antibacterial assay

The antibacterial potential of the optimum gel formulation was evaluated against methicillin-resistant *Staphylococcus aureus* (MRSA) and vancomycin-resistant Enterococcus (VRE), which were isolated from human samples and identified. The minimum inhibitory concentration (MIC) of the optimum formulation against tested microorganisms is measured by the microdilution technique.

### Identification of MRSA and VRE

Based on our previous studies, the identification of MRSA and VRE was performed according to their cultural characteristics and morphological and biochemical properties [31].

Bacterial samples isolated from human specimens were cultured in blood agar at 37 °C. After 24 h incubation, the Gram staining technique, coagulase test, mannitol agar cultivation, and DNase tests were carried out to identify *Staphylococcus aureus* (*S. aureus*) species. Then, *S. aureus* isolates were cultured on methicillin plates to consider the growing colonies as methicillin-resistant bacteria, MRSA.

Moreover, enterococcus species were identified based on enterococcus characteristics, including Gram staining technique, catalase, bile squalene hydrolysis, Pyrrolidonyl Arylamidase (PYR), hemolysis, optochin, growth in medium containing 6.5% sodium chloride and bacitracin susceptibility tests. Then, Enterococcus bacteria were cultured on vancomycin plates to consider the growing colonies as vancomycin-resistant bacteria, VRE.

### Micro dilution broth method

The antimicrobial activity of the selected formulation was assessed by microdilution methods, based on the clinical and laboratory standard institute (CLSI, 2019), against isolated resistant bacterial species, MRSA and VRE [32, 33]. For this purpose, serial dilution of selected formulation, formulation gel, the precipitate of probiotic, Vancomycin, and Methicillin was prepared in Mueller- Hinton broth at the concentration of 1000, 500, 250, 125, 62.5, 31.25, 16.12, 8 μg/mL in 96-well plates. Then, 10 µL of MRSA and VRE suspension were added to each well individually. After 24 h incubation at 37 °C, the optical density of all wells was measured at 600 nm by a microplate reader (Power Wave XS2, BioTek Instruments Inc., USA). In this study, the minimum concentration of each group inhibiting 90% MRSA and VRE growth was defined as the MIC value [34]. All treatments were carried out in triplicate.

### Statistical analysis

The results of the antibacterial assay were analyzed using IBM SPSS software. One-way ANOVA and Tukey's post hoc test were used to assess differences between the groups. All biological experiments were carried out in triplicate, and a statistically significant *P* value was considered ≤ 0.05.

## Results and discussion

The rise of bacterial resistance to various antimicrobial agents as a global health threat has attracted much attention to developing alternative methods in the last decades. As proposed by previous findings, probiotics could effectively cope with a wide range of resistant pathogens and improve human immunity. Accordingly, in the current study, five different formulations in two groups of water- and oil-based gels were designed, including a certain concentration of *L. rhamnosus* intended for probiotic use. In the first step of designing this formulation, we compare the viability of different lactobacilli strains, including *L. rhamnosus*, *Lactobacillus casei*, *Lactobacillus acidophilus* and *Lactobacillus fermentum* in the formulation; based on the results, *L. rhamnosus* was more sensitive to growth, so it was considered for further study. Additionally, the stability of *L. rhamnosus* was monitored in all groups for two weeks, and the most stable one was selected as the optimum gel formulation. The pharmaceutical aspects and the antibacterial activity of the optimum formulation were studied against MRSA and VRE, as some of the most resistant bacteria species to conventional treatment.

### Pharmaceutical assay

#### Formulation stability and viability of probiotic contents

**Fig. 1:**
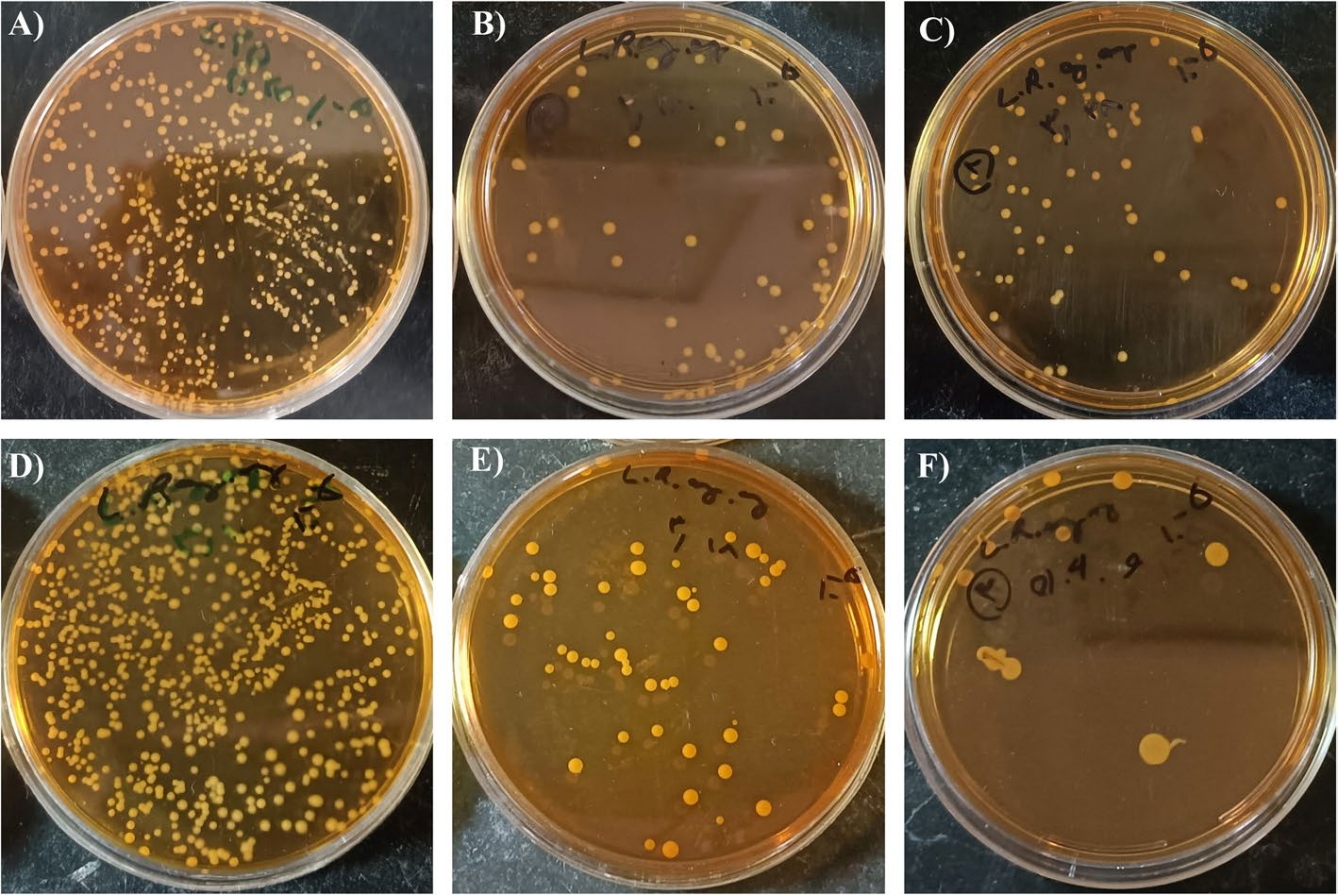
Bacterial stability of F4 formulation and F4 formulation solvent containing bacteria in 1:5 dilutions stored at 8 °C. Top and bottom rows are related to F4-solvent and F4 formulation respectively. **A** and **D**) day 1, **B** and **E**) day 7, and **C** and **F**) day 14

Bacterial stability of all the total designed formulations, formulation solvent containing bacteria, and gel bases was evaluated at 8 °C and reported as the CFU of bacteria on days 1, 7, and 14 in Table 2. The result indicated that the bacteria were stable in the water-based formulations (F1-F3) just for one week. In contrast, the oil-based formulations containing glycerol and olive oil demonstrated more stability, even an almost unaltered count in F4-solvent, after 14 days (Fig. 1). As pointed out in previous studies, glycerol, and olive oil reported antioxidant properties due to their phenolic contents and free radical-scavenging capacity that could help to preserve bacteria viability from the oxidation process and eventually improve *L. rhamnosus* stability in oil-based formulations [35–37]. Additionally, it seems that the presence of water in formulations could retard the growth of bacteria, which was probably responsible for the lower bacterial stability in water-based formulations [38]. Although formulation solvent groups of oil-based formulations (F4 and F5) revealed good bacterial stability, acceptable bacterial viability was reported in the total formulation of F4 after two weeks. Accordingly, F4 could act as the optimum probiotic formulation, which was chosen for another pharmaceutical assay.

**Table 2:** Bacterial stability of all designed formulations, bacterial CFU in days 1, 7, and 14 at 8 °C

Formulation	CFU count- Day 1 (CFU/ml)	CFU count- Day 7 (CFU/ml)	CFU count- Day 14 (CFU/ml)
F1	0.1 × 10^9^	1 × 10^5^	0
F1-Solvent	0.11 × 10^9^	0.26 × 10^6^	0
F1-Control	0	0	0
F2	0.45 × 10^8^	0.4 × 10^5^	0
F2-Solvent	0.84 × 10^8^	0.57 × 10^7^	0
F2-Control	0	0	0
F3	0.31 × 10^9^	0.3 × 10^5^	0
F3-Solvent	0.59 × 10^9^	0.23 × 10^6^	0
F3-Control	0	0	0
F4	0.18 × 10^9^	0.49 × 10^8^	1.1 × 10^7^
F4-Solvent	0.15 × 10^9^	0.78 × 10^8^	0.41 × 10^8^
F4-Control	0	0	0
F5	0.1 × 10^9^	0.5 × 10^5^	0.03 × 10^3^
F5-Solvent	0.14 × 10^9^	0.75 × 10^9^	0.5 × 10^4^
F5-Control	0	0	0

The results of the bacterial stability assessment showed that no bacterial viability was observed at 25 °C in the optimum formulation in line with previous findings [39], in contrast to CFU = 1.1 × 10^7^ at 8 °C, which emphasized the importance of storage temperature. Moreover, prior results indicated that the storage temperature and time significantly affect the viability of bacteria in lyophilized and suspension forms in formulation, as lyophilized bacteria showed more viability at 2–8 °C compared to 25 °C [40].

**Fig. 2:**
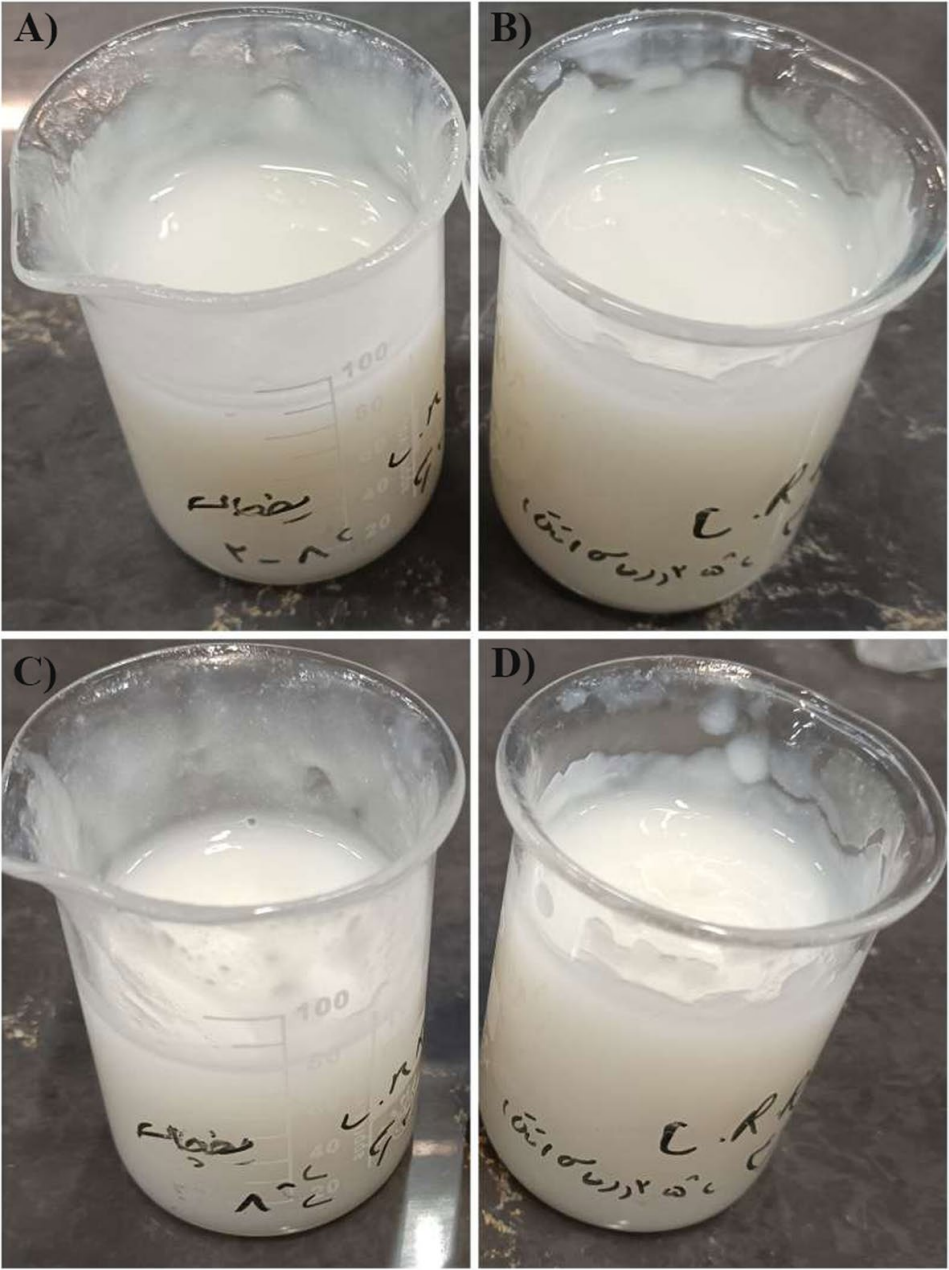
Physical stability of F4 formulation. **A**) day 1 at 8 °C, **B**) day 1 at 25 °C, **C)** day 1 at 8 °C and **D**) day 1 at 25 °C

Besides, the physical evaluation of the optimum formulation was monitored after 30 days at two temperatures of 8 °C and 25 °C. Our findings revealed no evidence of color, odor, volume changes, breaking of suspensions, crystal growth, or shrinking in the selected formulation (Fig. 2). Odor and color are significant factors in pharmaceutical products, which should not dramatically change shelf-life and storage. Odor changes could be induced by microbiological changes, oxidation of materials, the reaction of materials with each other, or reaction with the internal surface of the drug container, which should be considered in pharmaceutical studies.

### Homogeneity assays

Homogeneity is one of the main parameters for the uniformity and quality of semi-solid products. The F4 formulation exhibited high stability after centrifuging without any phase separation, which was categorized as very good in homogeneity classification.

### Rheological studies

**Fig. 3:**
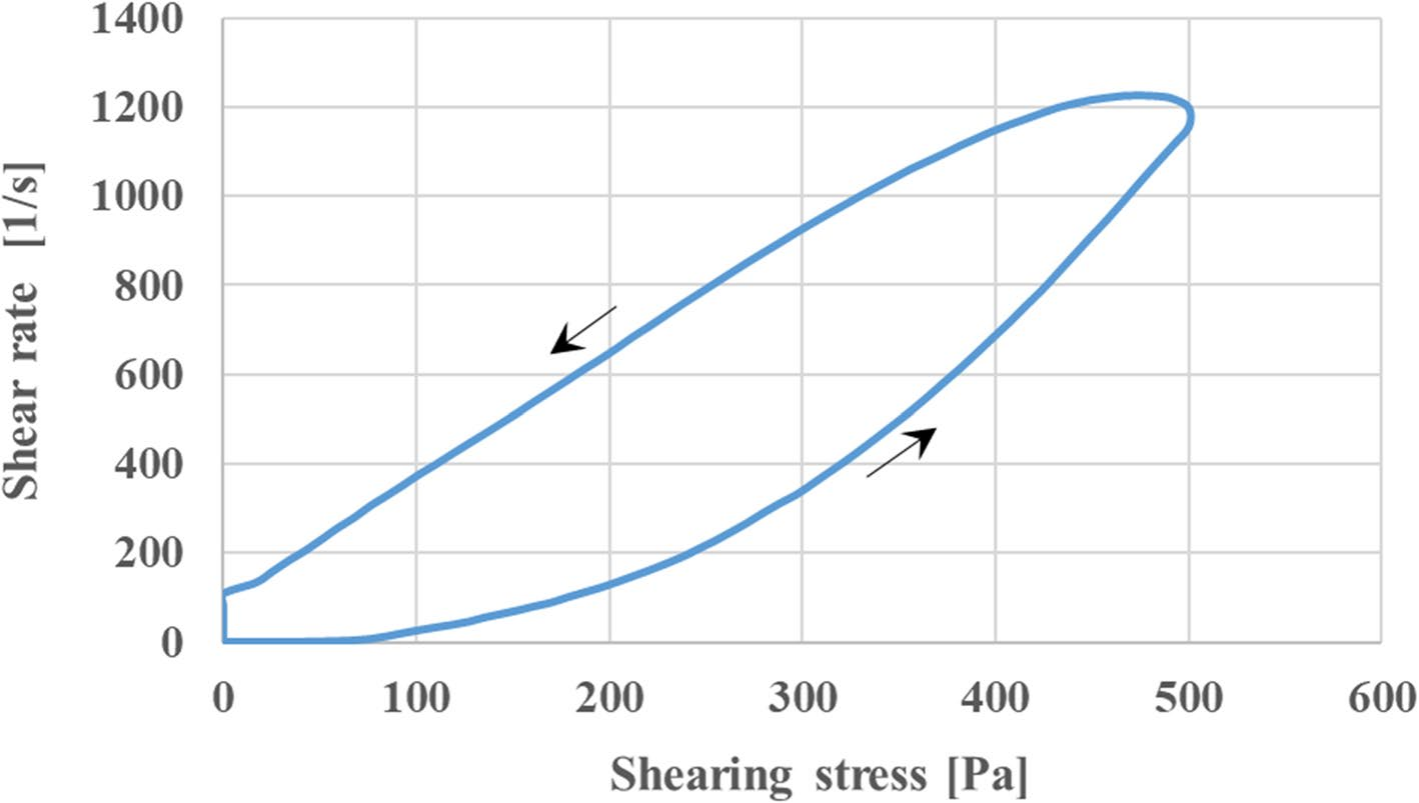
The shear rate vs. shear stress graph of F4 formulation at the temperature of 25 °C

Figure 3 shows the shear rates diagram versus shear stress to display rheological behavior in the selected formulation. According to this rheogram, with the increase in the shear stress, the shear rate increases, which illustrates the pseudo-plastic property of formulation.

Pseudo plastic property is a non-Newtonian behavior, which means that as soon as the shear stress is applied, the molecular arrangement changes from random to the linear form; this change allows internal resistance and apparent viscosity to be progressively reduced and flowability to expand[41, 42]. Furthermore, the graph shows the thixotropic properties of the formulation according to the hysteresis-loop area and the location of the descending curve that is above the ascending curve, which displays a decrease in viscosity over time at the constant shear rate [43].

According to previous studies, these properties in topical semi-solid products are ideal parameters for ease of use on the skin surface and bio-adhesive properties [44–46], and thixotropic behavior confirmed by hysteresis area is essential for physical stability of formulation [47, 48].

### Spreadability assays

**Fig. 4:**
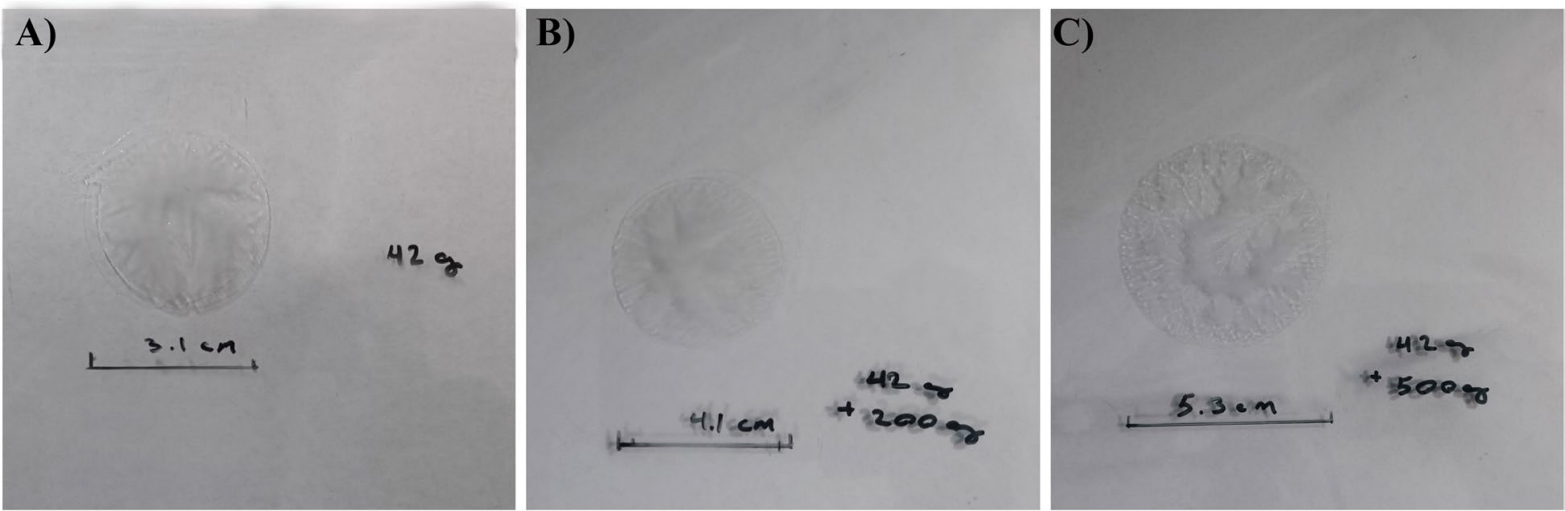
Spreadability of the optimum formulation under the force of **A**) 42 gr, **B**) 42 + 200 gr, and **C)** 42 + 500 gr

The spreadability of the gel is an essential property of uniform topical formulations, affecting patient compliance as an important factor in the therapeutic efficiency of products [49, 50]. In this study, the spreadability of F4 formulation was evaluated in force exertion of 42, 200 + 42, and 500 + 42 gr on triplicate samples, which are their mean sizes in Fig. 4 and Table 3. Our findings indicated that the spreadability behavior of the F4 gel formulation was acceptable since the circle diameter and area were in the normal range as a semi-solid product [51].

**Table 3:** Spreadability assay of optimum formulation, circle diameter, and circle area

**Weight (g)**	42	200 + 42	500 + 42
Circle diameter (mm)	31	41	53
Circle area (mm^2^)	754.385	1319.585	2205.065

### pH and Conductivity measurements

**Fig. 5:**
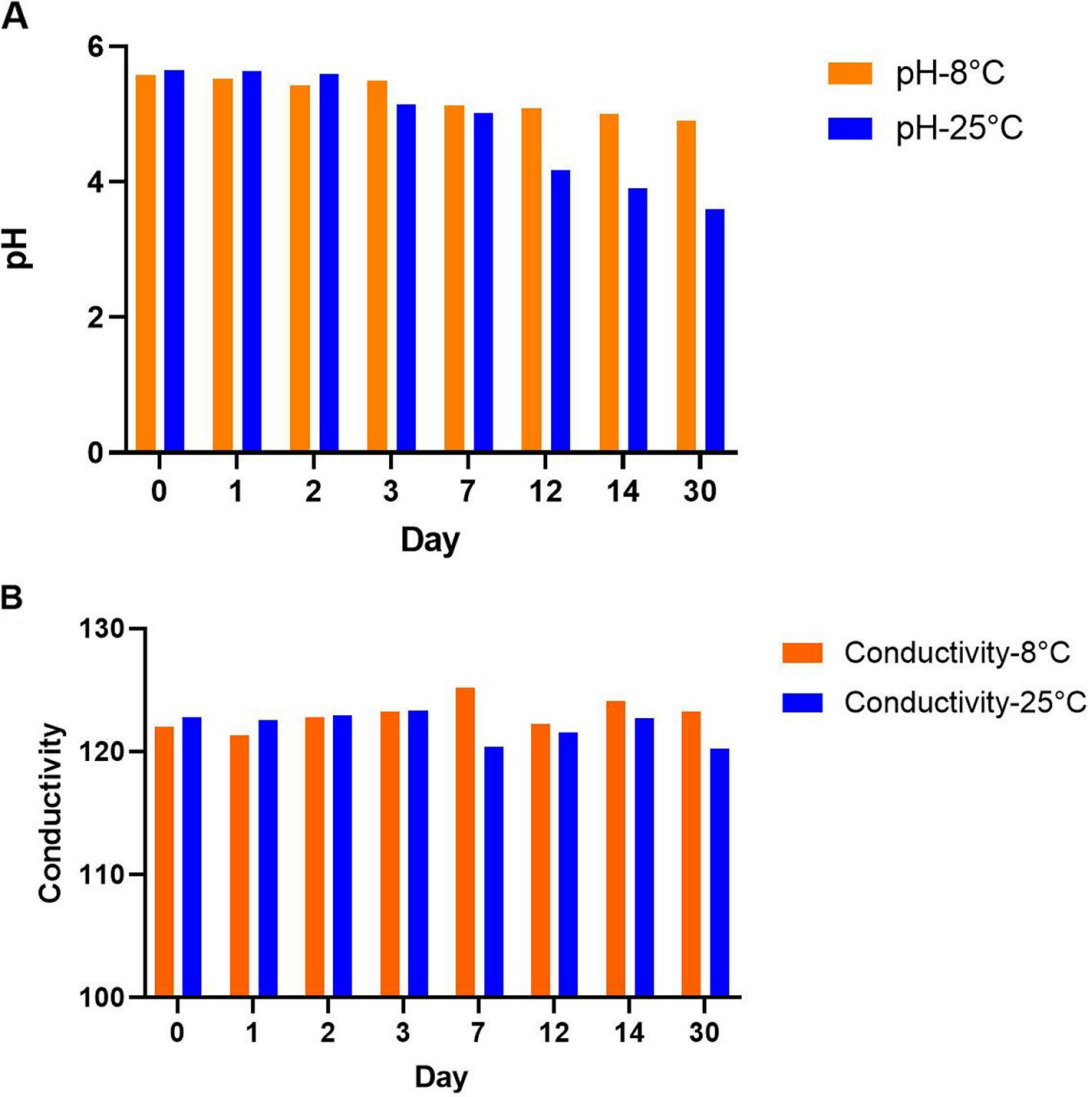
**A**) pH value and **B**) conductivity of F4 formulation stored at 8 and 25 °C for 30 days

Figure 5A displayed the pH value of the F4 formulation in two temperatures of 8 °C and 25 °C. The results showed that pH value was reduced at both 25 °C and 8 °C during the examination period, which reached 4.91 and 3.6 at 8 °C and 25 °C, respectively, due to the acidic nature of lactic acid bacteria, though this reduction was observed more at temperature of 25 °C. Since the desirable range of pH value for topical skin formulation without skin irritation was reported as 4.0–7.2 [48, 52], it appears that 8 °C is more effective and advisable for long-term storage of F4 formulation. Conductivity measurement of F4 formulation was approximately unaltered during the time, at the range of 120 to 125 mV at temperatures of 8 °C and 25 °C. (Fig. 5B). It is inferred that different temperatures showed the negligible influence on the conductivity of the design formulation during a month.

### Antibacterial assay

**Fig. 6:**
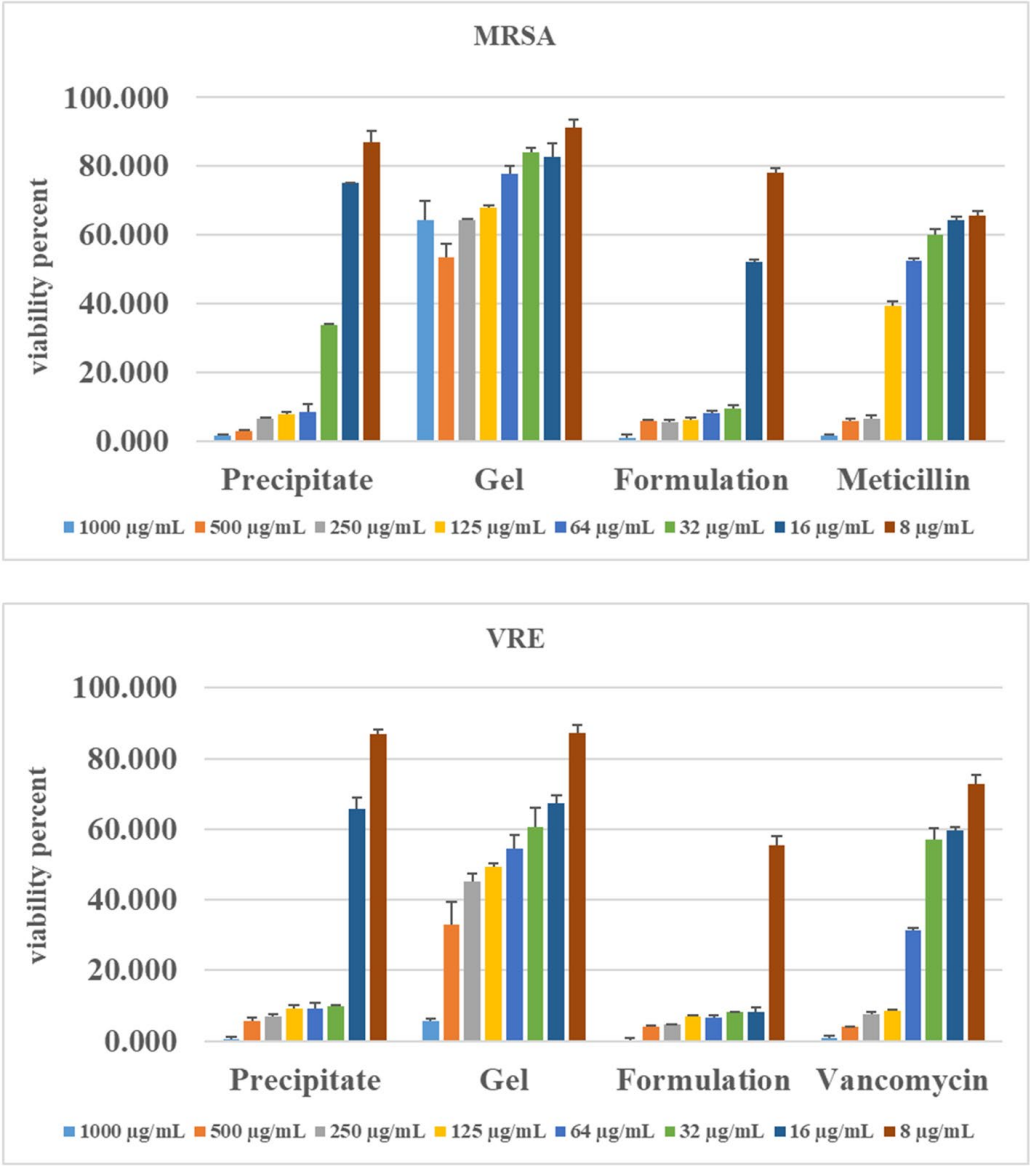
The viability percentage of MRSA and VRE treated by the F4 formulation, formulation gel, probiotic precipitate, Vancomycin, and Methicillin in different concentrations

As recent study interests are directed to the inhibition potential of the Lactobacillus family as effective probiotics for coping with various multi-drug resistant Gram-positive and -negative bacteria [53, 54], we evaluated the antibacterial potential of *L. rhamnosus* against two prevalent resistant Gram-positive bacteria as an active pharmaceutical ingredient (API) in the designed formulation. Figure 6 displayed the viability percentages of MRSA and VRE treated by the optimum formulation, formulation gel, probiotic precipitate, Vancomycin, and Methicillin. A dose-dependent manner was illustrated in bacterial growth inhibition results of all groups. The most and least bacterial inhibition activities were associated with the optimum formulation and formulation gel against MRSA and VRE (Table 4).

**Table 4:** MIC values of MRSA and VRE treated by the optimum formulation, formulation gel, probiotic precipitate, Vancomycin, and Methicillin in different concentrations

	**MRSA (µg/ml)**	**VRE (µg/ml)**
**Probiotic precipitate**	64	32
**Formulation gel**	> 1000	1000
**F4 Formulation**	32	16
**Methicillin**	250	….
**Vancomycin**	……	125

MIC values of the optimum formulation against MRSA and VRE were 32 and 16 μg/ml, while the formulation gel exhibited a MIC value of ≥ 1000 μg/ml in both groups. It confirmed that the formulation gel does not contribute to the antibacterial activity against tested pathogens, in line with the previous study [55], in which the antibacterial effect of six designed formulations containing *Lactobacillus plantarum* supernatants as API was evaluated against *Pseudomonas aeruginosa* as an opportunistic pathogen in chronic wounds. Moreover, Cabrera et al. found that *Lactobacillus plantarum* supernatants ( probiotic supernatants) presented more antipathogenic activities compared with that in the formulation [55], While our findings exhibited that the optimum formulation was more effective than probiotics individually. It can be inferred that the probiotic species is not the only practical factor in the antibacterial properties of the formulation. Although no inhibitory effect on the growth of pathogens was reported for the formulation gel, the greater antibacterial potential of the optimum formulation compared to probiotic precipitation could be assigned to the possible protective and synergetic effect of glycerol content of formulation, which added to the microbial inhibition potential of probiotics. As suggested in previous studies by Van Holm et al., glycerol could significantly strengthen the antibacterial properties of *Limosilactobacillus reuteri* in various oral biofilms in a synergetic synbiotic approach [56].

The antimicrobial potential of various probiotic species was evaluated against a wide range of pathogens, particularly hospital-associated microorganisms in the form of bacterial supernatants. As put forward by Chen et al., the probiotic supernatants, including *Lactobacillus fermentum*, *Bifidobacterium longum,* and *Bifidobacterium animalis*, displayed significant inhibitory properties against the growth of various MRSA strains as well as synergistic antibacterial effects in combination with bovine lactoferrin applied as a live food supplement [57]. *Lactobacillus acidophilus* was also suggested as an alternative medication for controlling MRSA infections [58]. The MIC value of 8 mg/ml was reported by Jameel et al*.* for the antibacterial activities of these beneficial probiotics against the 15 isolated MRSA species with significant inhibitory effects on biofilm formations, a primary determinant in the development and progression of bacterial resistance. Furthermore, *Saccharomyces boulardii,* applied as a probiotic yeast species to probiotic therapy of GI tract disorders have shown an effective role in reducing nosocomial transmission of VRE without remarkable side effects [59].

The evidence points to the significant anti-VRE and MRSA activity of optimum formulation and probiotic precipitate. However, VRE demonstrated more sensitivity than MRSA, which concurs well with previous findings. YÜKSEK et al. studied the antibacterial effects of cell-free supernatants of lactobacilli strains against MRSA, VRE, and Carbapenem-resistant Klebsiella. Their results indicated that lactobacilli species showed the most inhibitory impact on the growth of VRE in a dose-dependent manner [54].

Besides, the susceptibility of the probiotic bacteria in our formulation to Gentamicin and Cefoxitin antibiotics, the two common anti-microbial medications used to treat MRSA and VRE diseases, were evaluated for further assessment by disk diffusion methods based on CLSA 2020, which results are reported in supplementary files. The findings indicated that the probiotic bacteria showed no significant susceptibility to the tested antibiotics without notable zone inhibition (supplementary files). Moreover, given that these anti-microbial medications are administrated in parenteral route, it seems that there are no drug interactions between this topical formulation and common antibiotics in combination therapy and probiotic viability would be preserved.

Taken together, our findings have led us to propose that the optimum formulation could be a decent choice as the antimicrobial system for topical applications due to its significant bacterial inhibitory potentials and pharmaceutical properties—the optimum gel formulation based on glycerol provided desirable physical and probiotic stability, spreadability, and homogeneity. Moreover, its pseudo-plastic and thixotropic properties confer more stability and administration convenience.

Besides, the design formulation represented considerable antipathogenic behaviors against antibiotic-resistant species of MRSA and VRE as significant health-associated concerns. Notably, the oil base of the formulation offers additive effects on the antimicrobial potential of probiotics and the API of the formulation. It plays a protective role in improving its viability and functions as well. Bacterial immobilization of probiotics may be essential for survival during formulation processing [60, 61]. The studies showed that bacterial immobilization improved the survival and viability of Lactobacillus species [62]. Further research is also warranted to achieve a novel topical formulation containing effective probiotics for *in viv*o and clinical applications to control the growth and biofilm formation of multi-drug resistant opportunistic pathogens as antimicrobial dressing, particularly in chronic wounds, which have increased as a result of the rise in the aging population and the incidence of chronic diseases such as diabetes.

## Conclusion

Probiotic therapy is a reasonable way to cope with antibiotic resistance, one of the leading global health concerns. The current study offers *L. rhamnosus* as an effective API of the designed oil-based formulation. Our findings implied the inhibition efficiency of our developed formulation against MRSA and VRE growth, with even more VRE antimicrobial agent sensitivity. This formulation could be introduced as a good candidate for antibacterial agents in topical application, though further studies are needed to consider other aspects of clinical applications.

### Supplementary Information

**Additional file 1: Table S1. **Inhibition zone of formulation subjected to Gentamicin and Cefoxitin, Inhibition zone of diameter standard of Gentamicin and Cefoxitin based on CLSI 2020. **Figure S1. **Inhibition diameter zones obtained by paper disk diffusion method for formulation containing viable probiotic bacteria. (A): Cefoxitin (FOX); (B): Gentamicin (GEN).

## Data Availability

The datasets used and/or analyzed during the current study are available from the corresponding author on reasonable request.
