# Supplementary material for: Pharmacotechnical aspects of a stable probiotic formulation toward multidrug-resistance antibacterial activity: design and quality control
Source: BMC Complement Med Ther. 2023 Oct 31;23:391. doi: 10.1186/s12906-023-04224-0 (PMC10617127; doi:10.1186/s12906-023-04224-0)
Supplement: Supplementary file 1 — Additional file 1: Table S1. Inhibition zone of formulation subjected to Gentamicin and Cefoxitin, Inhibition zone of diameter standard of Gentamicin and Cefoxitin based on CLSI 2020. Figure S1. Inhibition diameter zones obtained by paper disk diffusion method for formulation containing viable probiotic bacteria. (A): Cefoxitin (FOX); (B): Gentamicin (GEN). [file 12906_2023_4224_MOESM1_ESM.pdf]

Pharmacotechnical aspects of a stable probiotic formulation toward multidrug-resistance antibacterial activity: design and quality control

Farkhonde Karimi [1,2], Amir Azadi [3,4], Navid Omidifar [1,5], Nima Montazeri – Najafabady [1,6], Fatemeh Mohammadi [1], Radmehr Kazemi [3], Ahmad Gholami [1,2,3*]

[1] Biotechnology Research Center, Shiraz University of Medical Sciences, Shiraz, Iran

[2] Department of Pharmaceutical Biotechnology, School of Pharmacy, Shiraz University of Medical Sciences, Shiraz, Iran

[3] Pharmaceutical Sciences Research Center, Shiraz University of Medical Sciences, Shiraz, Iran

[4] Department of Pharmaceutics, School of Pharmacy, Shiraz University of Medical Sciences, Shiraz, Iran

[5] Department of Pathology, School of Medicine, Shiraz University of Medical Sciences, Shiraz, Iran

[6] Endocrine and Metabolism Research Center, Shiraz University of Medical Sciences, Shiraz, Iran.

***Corresponding author:** gholami@sums.ac.ir. Tell: +987132426070

In this study, the susceptibility of the probiotic bacteria in the designed formulation to Gentamicin and Cefoxitin antibiotics, the two common anti-microbial medications used to treat MRSA and VRE diseases, were evaluated for further assessment by disk diffusion methods based on CLSA 2020, which results are reported in **Table S1** and **Figure S1**.

In the Disk Diffusion test, a thin formulation film containing viable probiotic bacteria applied on a plate is subjected to Gentamicin and Cefoxitin as the most conventional anti-microbial medications against MRSA and VRE. The inhibition Zone is an area around the antibiotic disk in which there is no bacterial growth. The diameter of the Inhibition Zone is an index of the susceptibility of the bacteria toward the antibiotics. The findings indicated that the probiotic bacteria showed no significant susceptibility to the tested antibiotics without notable zone inhibition.

Table S1. Inhibition zone of formulation subjected to Gentamicin and Cefoxitin, Inhibition zone of diameter standard of Gentamicin and Cefoxitin based on CLSI 2020.

Antibiotic	Inhibitory Zone diameter measured (mm)	Inhibition zone diameter standard (mm)		
	-	Resistance	Intermediate	Sensitive
Gentamicin	11	≤12	13-14	≥15
Cefoxitin (FOX)	R	≤21	-	≥22

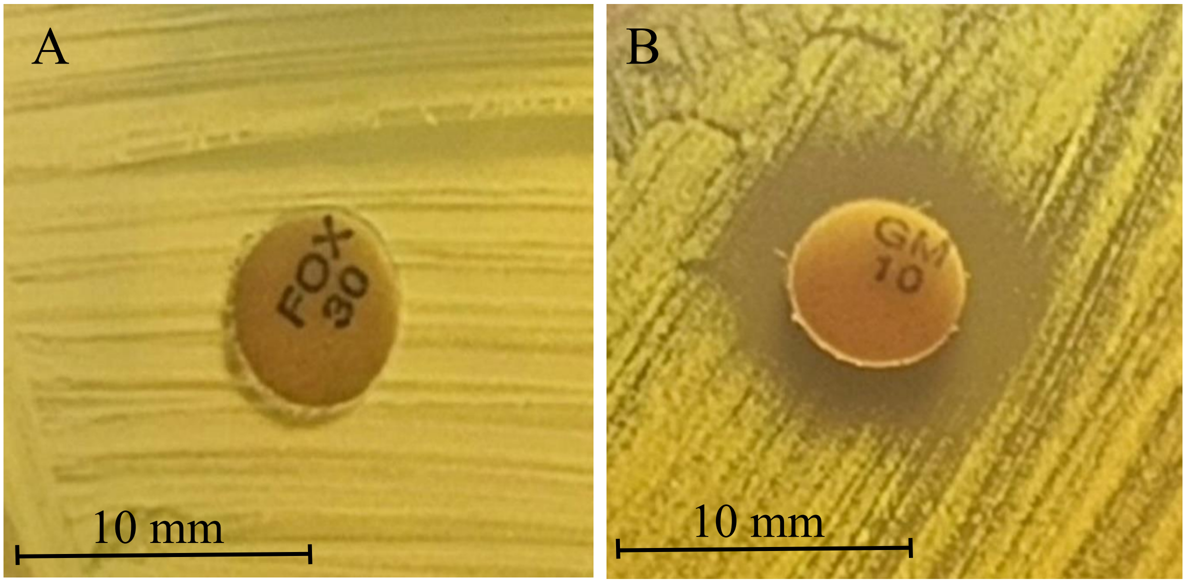

Figure S1. Inhibition diameter zones obtained by paper disk diffusion method for formulation containing viable probiotic bacteria. (**A**): Cefoxitin (FOX); (**B**): Gentamicin (GEN).
